# Cellularized, needle-deployed intracortical arrays for brain electrophysiology

**DOI:** 10.1557/s43579-026-01033-7

**Published:** 2026-08-19

**Authors:** Alexander J. Boys, Alejandro Carnicer Lombarte, Salim El-Hadwe, Sam Hilton, Chaeyeon Lee, Filip Wronowski, Tobias E. Naegele, Ana Fernandez-Villegas, Damiano G. Barone, Gabriele S. Kaminski Scherle, George G. Malliaras

**Affiliations:** 1 Department of Chemical Engineering & Biotechnology, University of Cambridge, Cambridge, CB1 0AS UK; 2 Thayer School of Engineering, Dartmouth College, Hanover, NH 03755 USA; 3 Department of Engineering, University of Cambridge, Cambridge, CB2 1PZ UK; 4 Department of Biomedical Engineering, City University of Hong Kong, Kowloon Tong, Hong Kong China; 5 Department of Clinical Neurosciences, University of Cambridge, Cambridge, CB2 0QQ UK; 6 Department of Neurosurgery, Houston Methodist, Houston, TX 77030 USA; 7 Neuroengineering Initiative, Rice University, Houston, TX 77030 USA

**Keywords:** Bioelectronic, Biomedical, Biomaterial

## Abstract

Bioelectronic medicine enables direct interfacing with native cell populations but remains limited by poor long-term integration and minimal interaction with non-electroactive cells. In contrast, regenerative medicine promotes tissue integration yet lacks bidirectional functionality. Here, we present a hybrid implantation methodology combining a tissue engineered cellularized hydrogel with a neural recording array for simultaneous delivery into the brain via needle placement. This approach enables co-localized transplantation of cells and electronics while minimizing surgical footprint. Post-implantation electrophysiological recordings show functional integration with surrounding neural tissue, demonstrating the feasibility of this platform for coupled bioelectronic sensing and regenerative capabilities.

**Graphical abstract:**

**Figure:**
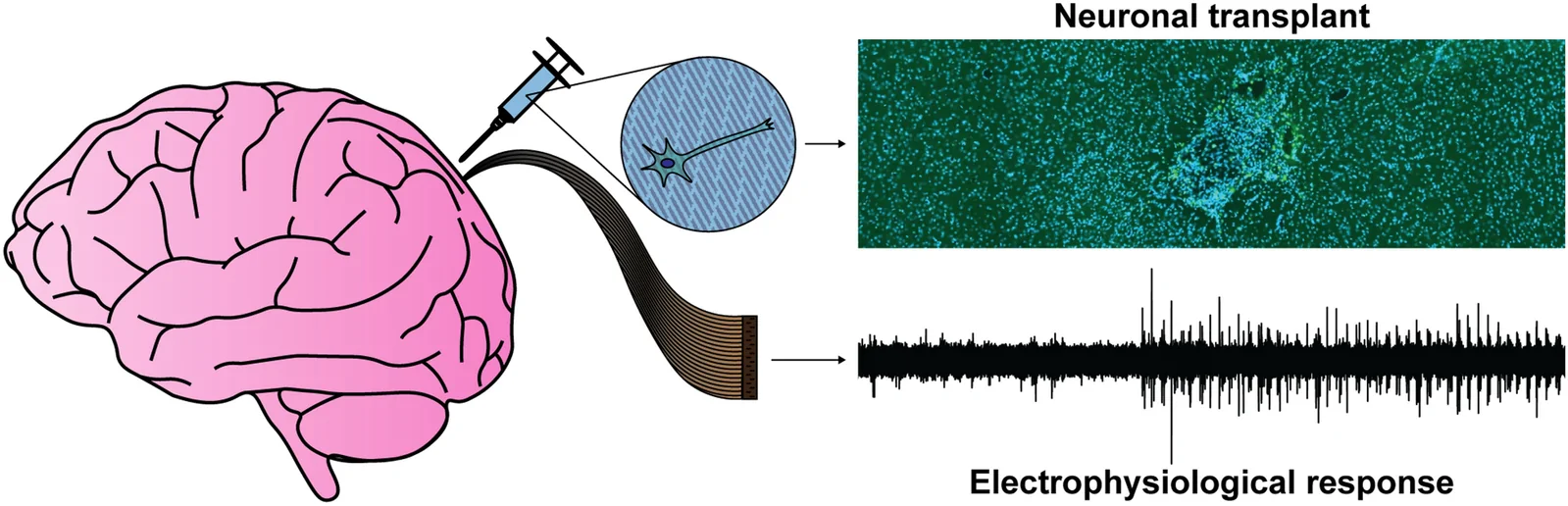

**Supplementary Information:**

The online version contains supplementary material available at https://doi.org/10.1557/s43579-026-01033-7.

## Introduction

Recent advancements in bioelectronic medicine have focused on the transplantation of live cells with an embedded device to act as an interfacial modality between the device and body.^[1–3]^ These so-called biohybrid devices^[4]^ are designed to solve multiple problems simultaneously, including lowering the inherent resolution limit afforded by our current generation of electrode materials and assisting in improved integration between the device and surrounding tissue. However, with these appealing prospects comes a variety of challenges. Many current bioelectronic devices use thin-film architectures due to their high-level of conformability, providing a robust interface with surrounding tissue. These devices are typically insulated using polymeric, dielectric and bioinert substrates, e.g., poly(dimethyl siloxane), poly(imide), and parylene-C.^[5]^ Growing and then transferring cells into the body on these highly flexible and generally inert substrates is challenging due to poor cell attachment and in-plane mechanical stress, which can result in cellular delamination from the substrate during handling. This general issue is also well-represented in the field of tissue engineering, where early work was focused on monolayer cell populations that poorly-represent the pericellular environments of native cell populations *in vivo*. Various strategies have been developed in the regenerative medicine field to avoid this issue, including the use of injectable extracellular matrices that contain viable cell populations in various tissues,^[6]^ including the brain.^[7]^ These systems essentially injection mold a cellularized “pre-gel” into a wound site,^[8]^ relying on *in situ* gelation to form an *in vivo* proto-tissue. Similar acellular concepts have been applied in some bioelectronic settings using *in situ* polymerization to produce conductive tracks inside of tissues.^[9–11]^ While these injectable-type strategies have been successfully deployed for some bioelectronic devices, these systems usually require some form of post-injection wire-bonding, due to the difficulty of driving backend connectors through a syringe cannula, to provide electronic access to the device. In this study, we present a method to simultaneously inject viable cell populations alongside bioelectronic devices, generating a fully integrated 3D cell population with embedded neural recording leads. This methodological study is presented to detail a means for simultaneously transplanting cells alongside functional bioelectronic devices.

## Methods

### Microfabrication

Bioelectronic devices were fabricated lithographically, as reported previously.^[12–15]^ Parylene-C dimer [(dichloro-p-cyclophane) Specialty Coating Systems, Indianapolis, IN, USA] was vapor deposited onto silicon wafers, using a vapor polymerization process [(SCS Labcoater) Specialty Coating Systems, Indianapolis, IN, USA] at 2 µm in thickness, forming the bottom of the device. Following deposition, wiring for gold tracks was photolithographically patterned [(MA/BA6 Mask Aligner) Süss Microtech, Garching, Germany] using an AZ 5214e photoresist [Microchemicals GmbH, Ulm, Germany] and a 1:4 ratio of AZ 351B developer [Microchemicals GmbH, Ulm, Germany] in water. A 10 nm Ti adhesion layer and a 100 nm Au layer were deposited using an electron-beam evaporator [(Lesker E-beam Evaporator) Kurt J. Lesker Company, Jefferson Hills, PA, USA] and patterned with a lift-off technique using acetone. A second layer of parylene-C was vapor polymerized at 2 µm, forming the top of the device. A micro90 [Cole Parmer, Vernon Hills, IL, USA] anti-adhesion layer was spin-coated on top of the device and a final 2 µm layer of parylene-C was deposited. The electrodes and connector were patterned using AZ 10XT 520cp photoresist [Microchemicals GmbH, Ulm, Germany] and a 1:4 ratio of AZ 400 k [Microchemicals GmbH, Ulm, Germany] developer in water. Reactive ion etching [(PlasmaPro 80) Oxford Instruments, Abingdon, UK] was used to expose the electrode and connector pads. Poly(ethylene dioxythiophene):poly(styrene sulfonate) (PEDOT:PSS) [(Clevios PH1000) Heraeus, Hanau, Germany], mixed with 5 v/v% ethylene glycol [Sigma Aldrich, St. Louis, MO, USA], 0.25 v/v% 4-dodecylbenzenesulfonic acid [Sigma Aldrich, St. Louis, MO, USA], and 1 v/v% (3-glycidyloxypropyl)trimethoxysilane [Sigma Aldrich, St. Louis, MO, USA], was spin-coated onto the wafer three times with a 100 °C soft bake between spins, patterned with a peel-off technique, and then hard baked at 130 °C for 1 h. Wafers were soaked in de-ionized water overnight. A final lithography step was performed using AZ 10XT photoresist and AZ 400 k developer as above to pattern the outline of the device, and the outline was generated through reactive ion etching. Devices were removed from the underlying substrate and bonded to flat flexible cables [(33-contact flex FFC cable) Molex 150140233, Lisle IL, USA] using anisotropic conductive film [(5 µm particulate] 3TFrontiers, Singapore] and a flip-chip bonder [(Finetech Bonder Fineplacer) Finetech GmbH, Berlin, Germany].

### Collagen extraction

Type I collagen was extracted from Sprague–Dawley rat tails, as previously reported.^[15–18]^ Briefly, tails were excised from deceased, skeletally-mature Sprague–Dawley rats [Charles River Laboratories, Kent, UK]. Skin was removed exposing the underlying tendon bundles. Adjacent disks were dislocated, allowing tendons to be dissected away from the tail. The tendon bundles were removed from the disk and placed into a 70% ethanol solution. Tendons were moved into a 0.1 v/v% acetic acid solution and left to solubilize for at least 48 h. Tendons were centrifuged and the supernatant was collected. The supernatant was lyophilized [(Labconco FreeZone) Labconco, Kansas City, MO, USA] and resuspended in 0.1 v/v% acetic acid for storage until use.

### Extraction of primary cortical neurons from P2-P4 rats

Primary neurons were extracted from rat pups [Charles River Laboratories, Kent, UK], as previously reported.^[19]^ Briefly, cortical tissue was isolated from P2 to P4 rat pups and digested in a solution consistent of Dulbecco’s modified eagle medium [Thermo Fisher Scientific UK, Loughborough, UK], 0.1% trypsin [Sigma Aldrich, St. Louis, MO, USA] and 0.05% DNAase [Sigma Aldrich, St. Louis, MO, USA] for 20 min in a CO_2_ incubator. Tissue was dissociated by trituration through 1 mL and 200 µL pipette tips before centrifugation at 600 rpm for 5 min. The supernatant was replaced with Neurobasal media consistent of 2% B27 [Thermo Fisher Scientific UK, Loughborough, UK] and 0.25% Glutamax [Thermo Fisher Scientific UK, Loughborough, UK], and the cell pellet was resuspended.

### Viability and morphological assays

For viability assays, extracted cortical neurons were plated into poly(styrene) culture flasks by injection under pressure through a 30G needle, the same gauge used for *in vivo work*. After 7 days in culture, cells were stained with a calcein AM and ethidium homodimer from a live/dead viability/cytotoxicity kit [Thermo Fisher, Waltham, MA, USA]. For viability assessment, cells were plated via injection under pressure through 25G and 30G needles and compared to pipette seeded cultures. Viability was assessed by manual counting across two sets of collected images. Neurons were also stained for βIII-tubulin and MAP2 and counterstained with Hoechst to confirm morphological characteristics.

### Surgical procedures

All procedures were performed in accordance with the UK Animals (Scientific Procedures) Act, 1986 and were approved by the animal welfare and ethical review body at the University of Cambridge. Procedures were performed under a project license by surgeons working with personal licenses, A. Carnicer-Lombarte, S. El-Hadwe, C. Lee, T.E. Naegele, and A.J. Boys, issued by the UK Home Office. Female Sprague–Dawley rats (200–250 g) were purchased [Charles River Laboratories UK, Kent, UK] and used for all animal work. An initial acute surgery was performed to assess device function in the brain. In this surgery, one rat was anaesthetized, and an implant was stereotaxically inserted unilaterally to a depth of 2500 µm into the hind limb sensorimotor cortex. The implant was connected to controller [(RHS Stim/Recording Controller) Intan Technologies, Los Angels, CA, USA] and a 32ch recording was performed with a 30 kHz sampling rat. The rat’s ipsilateral and contralateral paws were compressed by the surgeon (unblinded) at palpation pressure typically used to gauge hind limb reflex for anesthetic depth. The paws were alternately pressed. For chronic procedures, animals were anaesthetized for surgery using an isoflurane inhalant. Rats were given a pre-operative analgesic [(Rimadyl, 50 mg/mL stock) Zoetis, Parsippany-Troy Hills, NJ, USA] along with a saline injection. After surgery, rats received a post-operative analgesic [(Meloxicam, 1.5 mg/mL stock) Boehringer Ingelheim Ltd, Animal Health, Ingelheim, Germany] on days 1 and 2, post-surgery. For implantation, devices were bound to a 30G needle using poly(ethylene glycol) [Sigma Aldrich, St. Louis, MO, USA]. A craniectomy and a durotomy were performed. The needle with the device was placed into the hindlimb sensory motor cortex at a depth of 2.5 mm. Cellularized collagen gels were generated through a fibrillogenesis process, where collagen in acetic acid was neutralized and balanced for osmolarity and ionic strength to support cells using 1 N NaOH, 1 × phosphate buffered saline, and 10 × phosphate buffered saline, before primary cortical neurons (uncultured) in 1 × phosphate buffered saline were added to the pre-gel, as previously reported.^[15–18]^ This gelling solution containing 1 × 10^6^ cells/mL was injected at 10 µL/min into the brain using a syringe pump. The craniectomy window was sealed with medical-grade silicone (Kwik-Sil, World Precision Instruments, Sarasota, FL, USA), before the addition of bone cements [(Unifast Trad) GC America, Alsip, IL, USA; (Super Bond C&B) Prestige Dental Products Ltd, Bradford, UK] to create a head cap for wired access. Recordings were grounded using a cerebellar screw [(M0.8) US Micro Screw, Seattle, WA, USA] for electrophysiological recordings, backend wiring was housed inside a 3D-printed box, cemented onto the head of the rodent, and a transcranial screw was placed at the lambda for use as a ground. A total of *n* = 9 rats were implanted. Of these rats, *n* = 3 were used for electrophysiological analysis, *n* = 3 were used for immunofluorescent staining, and *n* = 3 were excluded due to exhibiting high mean impedances (> 1 MΩ at 1 kHz at Day 3 post-implantation) (Table I).

**Table I:** Description of rats used for different analyses.

Analysis	Day 1	Day 3	Day 7	Day 21
Electrophysiology	3 (continuous)	3 (continuous)	3 (continuous)	0
Immunofluorescence	0	0	1 (endpoint)	2 (endpoint)

Electrophysiological analyses were collected continuously as indicated, and immunofluorescence data were collected as an endpoint analysis.

### Electrophysiological recordings and analysis

Electrophysiological recordings were performed using a tethered connection to a headstage [(RHS 32-channel Stim/Recording Headstage) Intan Technologies, Los Angeles, CA, UA] and controller [(RHS Stim/Recording Controller) Intan Technologies, Los Angels, CA, USA] with data collected at a sample rate of 30 kHz. Data were analyzed in Matlab R2025a [Natick, MA, USA]. Electrophysiology data for each device and recording session was imported and filtered to both remove mains noise and isolate either high- (200–3000 Hz) or low- (1–200 Hz) frequency activity using a 4th order bandpass Butterworth filter. High-frequency activity was additionally processed by referencing to a common average signal (average signal across all electrodes subtracted from every electrode) to isolate electrical activity local to every electrode. For both high and low frequency bands, signal amplitude at every electrode in the form of the average root-mean-squared value of the signal was calculated. RMS amplitude was compared for the same electrodes on the device across multiple timepoints using either a two-sample Student’s *t*-test (2 timepoints) or an ANOVA and Tukey post hoc test (> 2 timepoints).

### Immunofluorescence staining

For immunofluorescence staining, rats were perfused with 4% paraformaldehyde. The brain was removed with the skull intact and fixed overnight at 4°C. Afterward, the tissue was incubated in 30% sucrose in phosphate buffered saline (PBS) for approximately 48 h. A cryostat was used to cut 20–40 µm sections from the tissue, and these sections were mounted onto slides. Following fixation, slides were washed three times in PBS to remove cryoprotectant and then incubated for 1 h in PBS with 0.3% triton x-100. Afterward slides were incubated for 1 h at room temperature in 5% normal donkey serum in PBS. Slides were incubated with primary antibody [(1:500 GFAP, ab7260) Abcam, Cambridge, UK; (1:500 CD11b + 1:500 CD11c, ab33827) Abcam, Cambridge, UK; (1:500 NeuN, ab104224) Abcam, Cambridge, UK] overnight at 4 °C and then washed three times with PBS. Following this, slides were incubated for 2 h at room temperature with secondary antibodies [(anti-mouse Alexa Fluor 488) Abcam, Cambridge, UK; (anti-rabbit Alexa Fluor 555) Abcam, Cambridge, UK]. Finally, slides were stained with DAPI [(NBP-31156) Novus, Centennial, CO, USA] at a 1:5000 dilution in PBS for 10 min and stored at 4 °C for imaging. Images were collected either using a confocal microscope [(Zeiss LSM 800), Zeiss, Badem-Württemberg, Germany] using a slide scanner [(Zeiss AxioScan) Zeiss, Baden-Württemberg, Germany].

## Results and discussion

### Deployment strategy

**Figure 1:**
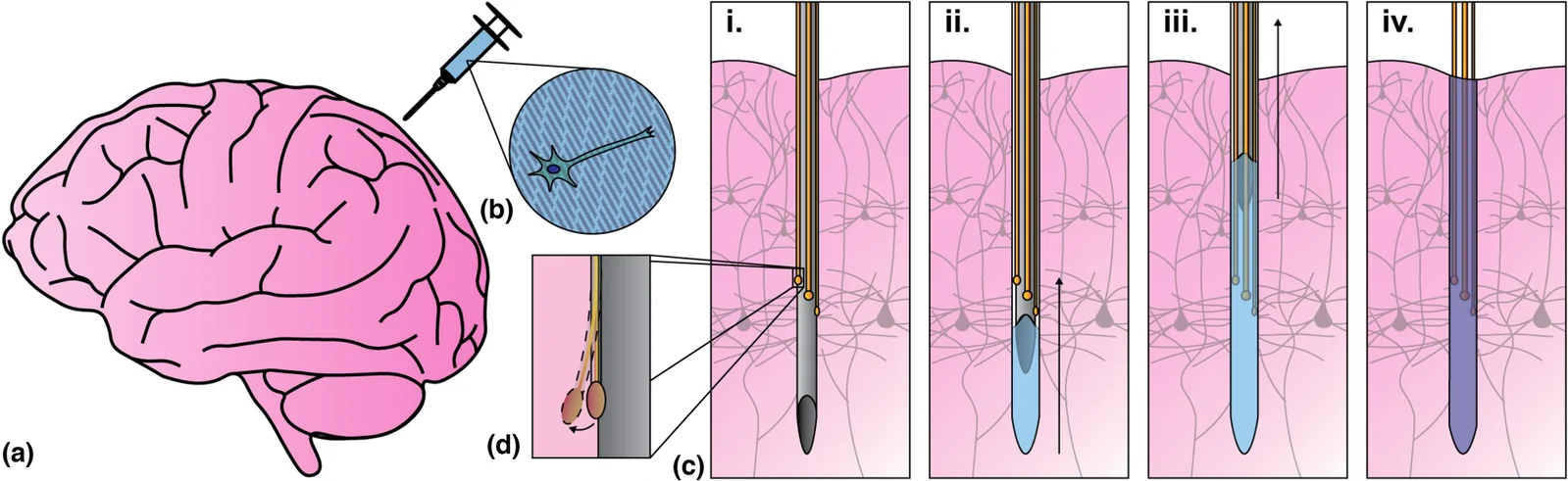
(a) Schematic of injection strategy, where (b) a syringe loaded with a cellularized pre-gel solution is used to place a bioelectronic device into the cortex and then backfill the placement cavity with the tissue engineered gel system. (c) Injection scheme, where (d) the bioelectronic device is designed for timed release from the needle to occur after insertion into the cortex. (i) Needle loaded with device and cellularized pre-gel is placed into the cortex. (ii) As the needle is retracted from the insertion site, the cellularized pre-gel is injected, backfilling the cavity made by the needle. (iii) The adhesive holding the implant on the needle dissolves, allowing the implant's release into the insertion site. (iv) The cellularized pre-gel gels, creating a tissue-like structure containing the bioelectronic leads inside the insertion site. In this study, needles were inserted to a depth of 2500 µm.

We developed a bioelectronic device system compatible with syringe injection, enabling its implantation alongside injected cell populations contained within a self-assembled extracellular matrix (Fig. 1). For this study, we demonstrate our devices as intracortical implants for accessing sensorimotor information. To enable our deployment mechanism, we utilized a prior implant design possessing a minimalistic tissue interface.^[15]^ This implant consists of a series of 32 individually-articulated recording leads, each 8 × 4 µm in cross-section and between 500 and 2500 µm in length. To implant these devices, we bound the leads to a 30G needle using PEG as a bioresorbable adhesive. Adhesion was performed by dipping the needle with the device in its vicinity into a PEG solution, binding each lead to the exterior of the cannulated needle. By using PEG as an adhesive, we could place the needle into the target tissue, where the baseline tissue hydration would dissolve the PEG adhesive, allowing the needle to be removed while the implant remains embedded in the tissue. This general strategy has been used in other instances alongside micro-scale wires, which have been used as stiff placement shuttles for positioning flexible devices into soft tissues, such as the brain.^[15,20]^ We specifically used PEG as a release-method in the brain in our prior work,^[15]^ indicating that this mechanism is compatible with brain implantation. For our system, once adhered to the needle, the cannula can be used to inject a cellularized pre-gel. Specifically, we used a syringe pump to inject the gel while retracting the needle from the tissue, thereby backfilling the cavity with the cellularized gel and implants.

### Component development

#### Microfabrication and acute testing

**Figure 2:**
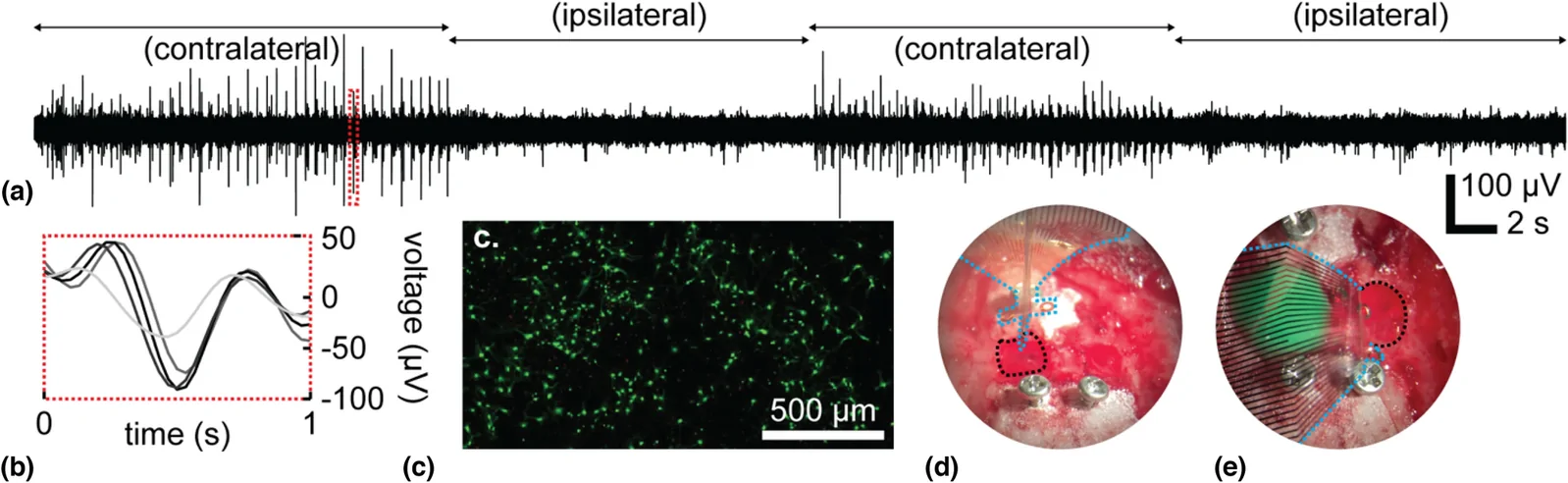
(a) Voltage trace demonstrating electrophysiological function of recording device as a high-resolution recording implant. Device were inserted into the sensorimotor cortex for hindlimb using 50 µm tungsten wire. As the rat’s contralateral paw is squeezed, spiking activity is observed, whereas when the ipsilateral paw is squeezed, no spiking activity is observed. (b) Traces show temporal synchronization across electrodes within a singular tetrode, indicating validity of spiking data format, Red dashed box in (a) indicates analyzed portion of trace. Coloring of traces is arbitrary to enable visualization. (c) Live/dead stain showing primary neurons injected at high pressure through syringe into culture plate at 7 days, indicative of cellular survival through transplant procedure. (d) Image showing device mounted on needle immediately prior to placement and (e) after needle removal. Device is outlined in blue and cranial window is outlined in black. Green silicone is visible, which was used to seal contralateral insertion, possessing no gel. Images in (d and e) were collected stereoscopically with a large depth-of-field. To provide scaling for the image, the visible screw is M0.8 with a screw head diameter of ~ 1.3 mm.

We constructed the flexible bioelectronic devices using cleanroom-based photolithographic methods to generate devices with a minimalistic bioelectronic interface. The use of a minimalistic interface is a critical component for this system to facilitate gel integration with surrounding tissue. These devices were of the same design as a previous device construction.^[15]^ After fabrication, we initially tested this device in an acute *in vivo* setting to confirm its efficacy as a valid recording system. We implanted the device into the right sensorimotor cortex for hind limb in a rat. Once implanted, we applied a stimulus to rat’s left and right paws, finding that the data displays high-frequency activity with a spiking-like waveform that is present on all electrodes in a tetrode only when the contralateral paw is palpated, consistent with what would be expected for a putative single unit recording [Fig. 2(a) and (b)]. It is notable that due to the articulated lead design, the tetrode format is somewhat variable, as the leads can move relative to one another during placement, meaning that a standardized tetrode geometry is not ensured. The tetrode geometry was chosen to provide electrode redundancy for single unit isolation if desired.

#### Cellular extraction and testing

For extracellular matrix, we chose to use rat tail-derived Type I collagen, which we have used in the past as an injectable cellularizable medium. Type I collagen is fibrillar and is soluble at low pH. Upon raising the pH, cells can be mixed into the gelling solution, and the collagen will self-assemble in a fibrillar, homogeneously seeded gel. For a cellular population, we used primary neurons extracted from P2-P4 rat pups. We chose this cell population for its relative plasticity, providing a means for potential integration with surrounding brain tissue. This postnatal neural population was chosen arbitrarily over embryonic neurons. To test the deployment strategy rather than immune response, we extracted collagen from mature Sprague–Dawley rats and neurons from Sprague–Dawley rat pups. Sprague–Dawley rats are outbred but are still a relatively well-conserved strain, meaning that we would not expect a standard allogeneic immune response in this system. To test the viability of our cells when subjected to this injection system, we injected a population of these neurons at maximal pressure through a 30G needle into a culture plate and performed live/dead staining after one week, finding viability [Fig. 2(c)] comparable to cells injected under maximal pressure through a 25G needle and to cells plated via pipetting [Fig. S1(a–g)]. To assess cell morphology, neurons were also stained for βIII-tubulin and MAP2 with a Hoechst nuclear counterstain, confirming neuronal characteristics [Fig. S1(h–m)].

#### Cadaveric demonstration

Finally, to determine the efficacy of this implantation mechanism, we performed injections using collagen gel in rodent cadavers to confirm that the gel could be injected intracortically. For this experiment, we combined collagen with rhodamine B dye and injected the dye mixture into the cortex (Video S1). We noted consistent filling of the defect site with eventual efflux of the gel after a volume of 14µL. These tests enabled us to set our implantation parameters for *in vivo* testing.

### Device implantation and testing

After parameter optimization, we implanted our devices (Fig. S2) in Sprague–Dawley rats using the same implantation scheme. To place the probe, we stereotaxically inserted the needle into the cortex and released the probe backend from the frame [Fig. 2(d)]. We used a syringe pump to inject cellularized pre-gel into the brain during probe extraction, leaving the probe and gel in the cortex [Fig. 2(e)]. After probe and gel were injected, we sealed the defect with silicone followed by bone cement, and, for electrophysiological access, used a 3D-printed head cap for tethered access. Devices were placed into the sensorimotor cortex for limb motion. We note that while Type I collagen is not native to brain tissue, the brain does contain type IV collagen and associated collagenases.^[21]^

### Device electrophysiological performance and characterization

**Figure 3:**
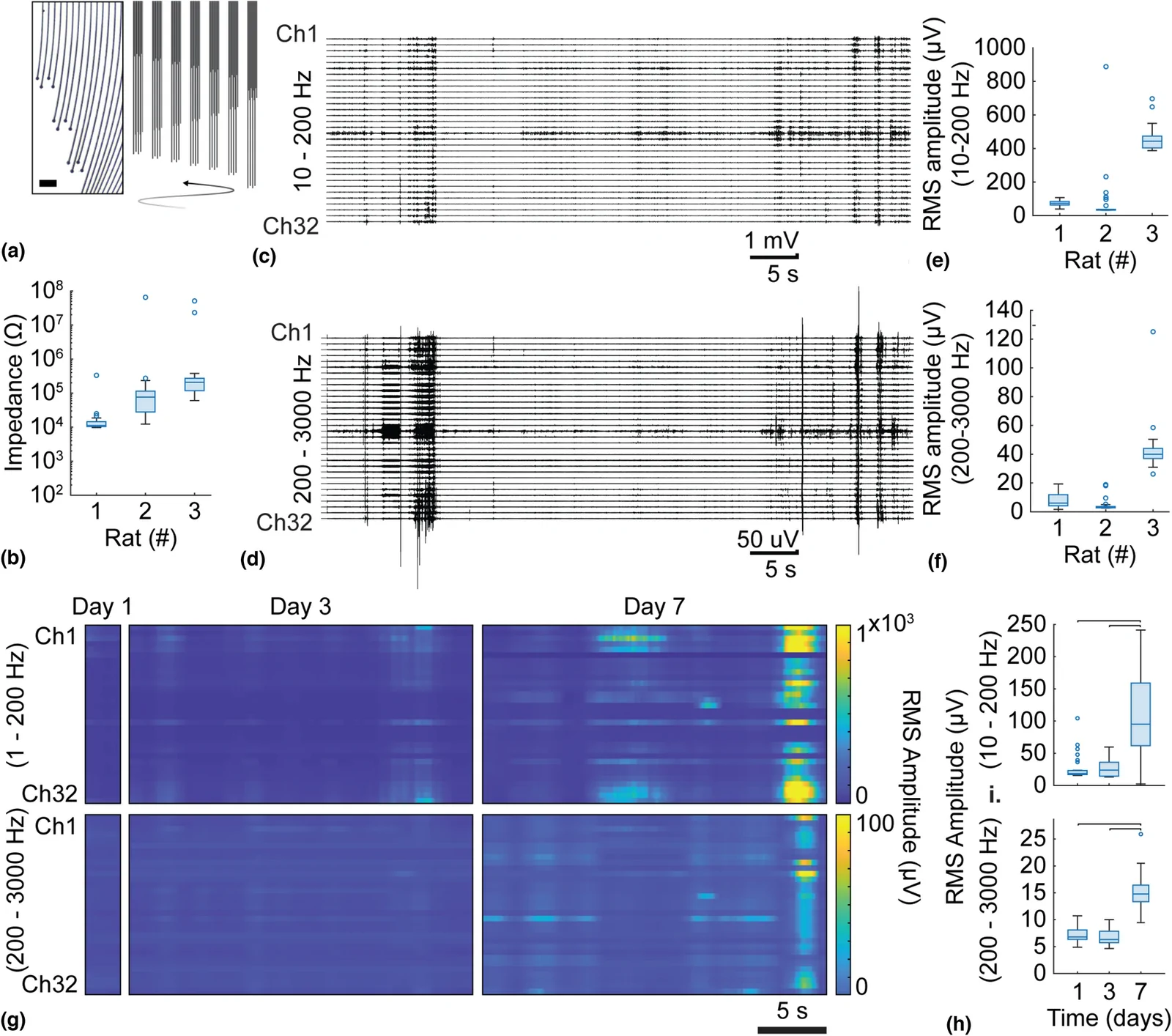
(a) Image of device (left) and accompanying diagram (right) of approximate recording layout configuration, where electrodes spiral around a central axis, which originally contains the needle, at varying heights in a tetrode format. Scale bar is 100 µm. (b) Boxplot of electrode impedance across three rats. Electrodes with impedances > 10 ΜΩ are considered non-functional and not shown. Recording from 32 electrodes of an implanted device at Day 3 post-implantation in freely-moving rats. Multichannel recordings for both (c) low- and (d) high-frequency bands are shown. Box plots showing root-mean-squared (RMS) amplitude for (e) low- and (f) high-frequency bands for each rat at Day 3 implantation. Heatmap quantification of recording RMS amplitude across implant channels for a week of implantation (at Days 1, 3, and 7 post-implantation) for one rat (Rat#1) for both low (top) and high (bottom) frequency bands. Boxplots of average RMS amplitude for all electrodes in a device across a week post-implantation, for one rat (Rat#1: (h) high and (i) low frequency). Brackets indicate *p* < 0.0001 from Tukey multiple comparison test.

To evaluate device function and integration into the brain, we carried out electrophysiological recordings of awake rats while an experimenter pressed on their right and left front paws in an alternating fashion, over 30 s intervals. Functional devices were classified based on impedance and shown to perform neural recordings in awake animals three days post-implantation [Fig. 3(a–f)]. For visualization, traces were filtered for analysis at 1–200 Hz (local field potential-type activity) and from 200–3000 Hz (compound/single unit-type activity). Impedance analysis of all electrodes showed that 96.88% of these remained functional 3 days post-implantation, which we defined as having impedances below 10 MΩ [Fig. 3(b), 100.11 ± 32.50 kΩ for all functional electrodes, mean ± standard deviation], similarly indicating that devices had survived the chronic implantation procedure and were able to operate correctly. To identify whether the combination of device and transplanted cells were able to integrate into the host brain, we tracked changes in signal amplitude over seven days of implantation in two animals. We observed that signal amplitude reliably and significantly increased in these animals [Fig. 3(g–l), across days 1, 3 and 7 post-implantation, *p* < 0.0001 for both rats and both frequency ranges*,* one-way ANOVA], supporting that both transplanted and local host neurons were integrating and producing the recorded higher amplitude signals.

**Figure 4:**
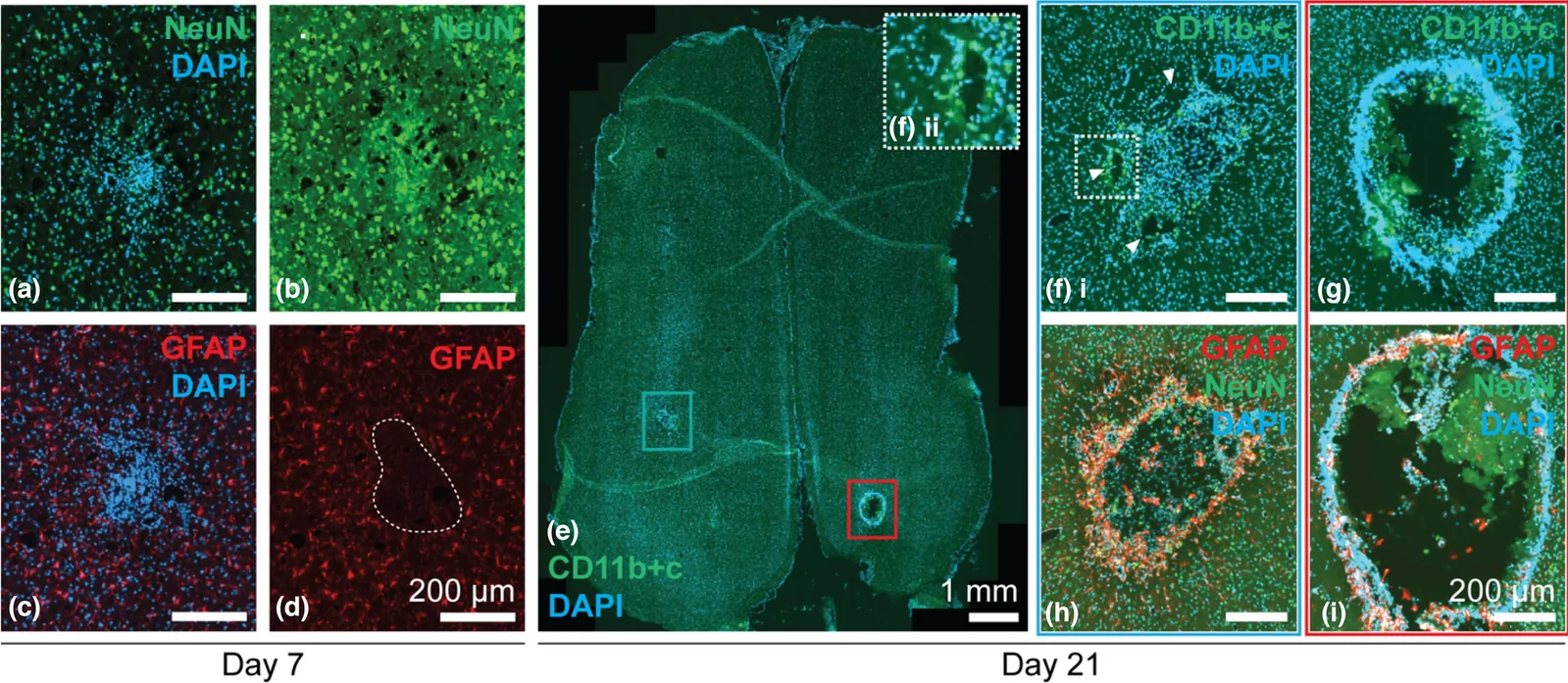
(a) Immunofluorescent images of gel and implant injection site at Day 7 post-implantation, stained for cell nuclei (DAPI—blue) and neurons (NeuN—green). (b) Same image from (a) but with nuclear staining removed and contrast from neuron stain is saturated to show tissue morphology around insertion site. (c) Images from Day 7 of stain for cell nuclei (DAPI—blue) and astrocytes (GFAP red). (d) Same image from (c) with nuclear stain removed to show lack of astrocytes within insertion site, indicative of neuron transplant. Insertion site is outlined. (e) Image of horizontal brain section at Day 21 post-implantation showing insertion site containing cellularized gel and device (blue) and device only (red), visualized through staining for cell nuclei (DAPI—blue) and activated microglia (CD11b, CD11c—green). Images of cell nuclei (DAPI—blue) and activated microglia (CD11b, CD11c -green) for (f) (i) gel and device and g. device only at Day 21. Arrows in (f) (i) indicate perforations, and inset (white box) in (f) (ii) shows activated microglia near perforation. View field is ~ 20 × 20 µm. Images of cell nuclei (DAPI -blue), astrocytes (GFAP—red), and neurons (NeuN—green) for (h) gel and device and (i) device only at Day 21.

Immunofluorescence data were collected for rats at Days 7 (*n* = 1) and 21 (*n* = 2). Images show a viable cell population within insertion site at Day 7, which shows positive staining for NeuN, indicative of live neurons within insertion site [Fig. 4(a)]. Little evidence of a defined interface is present at this timepoint. Perforations around insertion site show possible location of implant leads [Fig. 4(b)]. Staining for astrocytes shows no astrocytes within insertion site (consistent with neural cell extraction) nor any astrocytic infiltration into the insertion site interior [Fig. 4(c) and (d)]. At Day 21, insertion site maintains integrative morphology and shows minimal microglial activation, particular versus sham control, which contains the device but no cellularized gel [Figs. 4(e–g), S3, and S4]. Perforations are visible around the periphery of the insertion, indicating possible lead sites. Some activated microglia are visible near perforations [Fig. 4(f)]. Some neural staining is visible within insertion site and astrocytes are visible around periphery of insertion site [Fig. 4(h and i)]. Interestingly, astrocytes are also visible within insertion site, indicating astrocytic infiltration from native tissue into tissue engineered gel interior.

Overall, these results indicate that the devices we developed are able to remain functional after being implanted into the brain and perform electrophysiological recordings in a stable manner in chronic, awake conditions. Furthermore, these results also support that the injected transplant and surrounding tissue stabilize over the first week following surgery, leading to an increase in signal amplitude which the injectable devices are able to record. This system shows a mechanism to transplant viable cells alongside functional bioelectronics into the brain. These methods are viable for expansion into other areas of the body, presenting a new technique for the simultaneous transplant of bioelectronic regenerative medicine.

## Supplementary Information

Below is the link to the electronic supplementary material.

Supplementary file1 (DOCX 6073 KB)

Supplementary file2 (MOV 7836 KB)

## Data Availability

The data that supports the findings of this study are available from the corresponding author on reasonable request.
