# Supplementary material for: Cellularized, needle-deployed intracortical arrays for brain electrophysiology
Source: MRS Commun. 2026 Aug 19;16(5):1272–9. doi: 10.1557/s43579-026-01033-7 (PMC13743597; doi:10.1557/s43579-026-01033-7)
Supplement: Supplementary file 1 [file 43579_2026_1033_MOESM1_ESM.docx]

**Supplemental Information**

**Cellularized, needle-deployed intracortical arrays for brain electrophysiology**

Alexander J. Boys,^1,2^ Alejandro Carnicer Lombarte,^3,4^ Salim El-Hadwe,^3,5^ Sam Hilton,^3^ Chaeyeon Lee,^3^ Filip Wronowski,^3^ Tobias E. Naegele,^3^ Ana Fernandez-Villegas,^1^ Damiano G. Barone,^3,5,6,7^ Gabriele S. Kaminski Scherle,^1^ George G. Malliaras^3,^*

^1^ Department of Chemical Engineering & Biotechnology, University of Cambridge, Cambridge, CB1 0AS, UK

^2^ Thayer School of Engineering, Dartmouth College, Hanover, NH 03755, USA

^3^ Department of Engineering, University of Cambridge, Cambridge, CB2 1PZ, UK

^4^ Department of Biomedical Engineering, City University of Hong Kong, Kowloon Tong, Hong Kong

^5^ Department of Clinical Neurosciences, University of Cambridge, Cambridge, CB2 0QQ, UK

^6^ Department of Neurosurgery, Houston Methodist, Houston, TX, 77030, USA

^7^ Neuroengineering Initiative, Rice University, Houston, TX, 77030, USA

*Corresponding author: gm603@cam.ac.uk

**Video S1.** Video showing injection of rhodamine and collagen pre-gel solution into cadaveric cortex. Prior to efflux, the brain swells, providing evidence that the injected fluid fills the cavity made by the needle. For surgical procedures, injections were performed via syringe pump to reduce pressure on brain tissue.
